# Supplementary material for: Expression Profiles of Housekeeping Genes and Tissue-Specific Genes in Different Tissues of Chinese Sturgeon (Acipenser sinensis)
Source: Animals (Basel). 2024 Nov 21;14(23):3357. doi: 10.3390/ani14233357 (PMC11639794; doi:10.3390/ani14233357)
Supplement: Supplementary file 1 [file animals-14-03357-s001.zip › Figure S4.pdf]

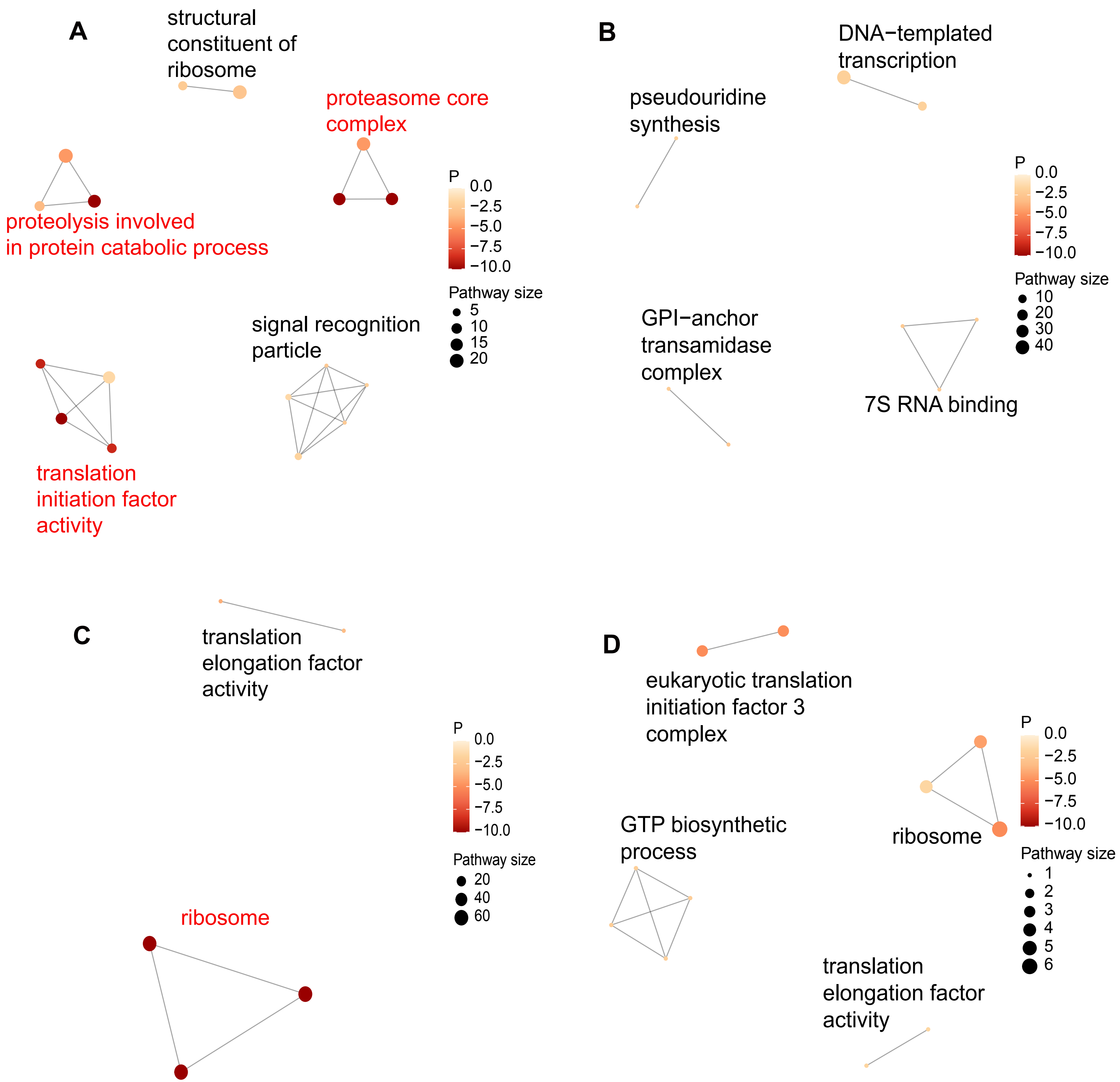
A
structural constituent of ribosome
proteasome core complex
proteolysis involved in protein catabolic process
signal recognition particle
translation initiation factor activity
P
0.0
−2.5
−5.0
−7.5
−10.0
Pathway size
5
10
15
20
B
DNA−templated transcription
pseudouridine synthesis
GPI−anchor transamidase complex
7S RNA binding
P
0.0
−2.5
−5.0
−7.5
−10.0
Pathway size
10
20
30
40
C
translation elongation factor activity
ribosome
P
0.0
−2.5
−5.0
−7.5
−10.0
Pathway size
20
40
60
D
eukaryotic translation initiation factor 3 complex
GTP biosynthetic process
ribosome
translation elongation factor activity
P
0.0
−2.5
−5.0
−7.5
−10.0
Pathway size
1
2
3
4
5
6
