# Supplementary material for: Expression Profiles of Housekeeping Genes and Tissue-Specific Genes in Different Tissues of Chinese Sturgeon (Acipenser sinensis)
Source: Animals (Basel). 2024 Nov 21;14(23):3357. doi: 10.3390/ani14233357 (PMC11639794; doi:10.3390/ani14233357)
Supplement: Supplementary file 1 [file animals-14-03357-s001.zip › Figure S6.pdf]

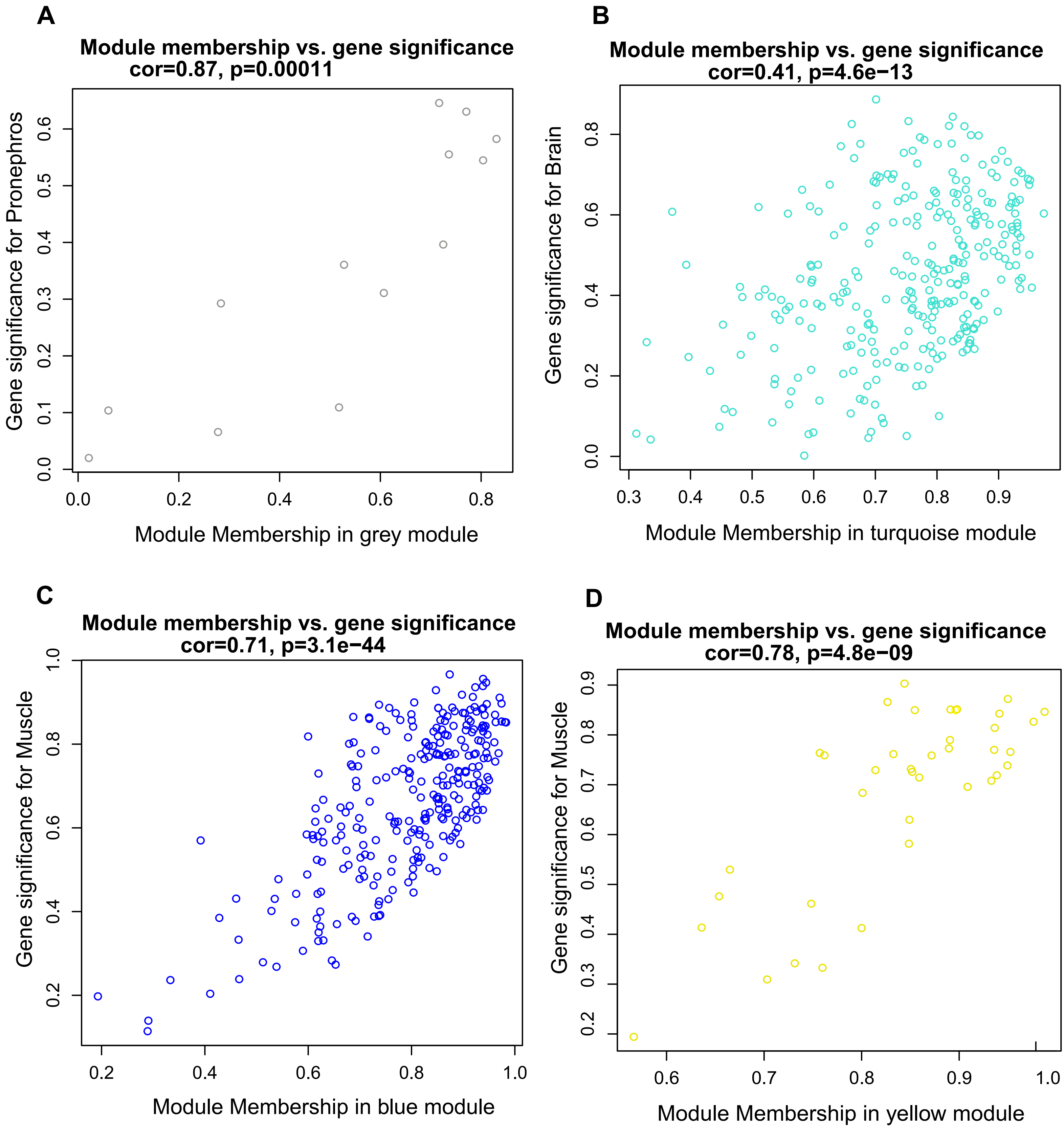
A
Module membership vs. gene significance
cor=0.87, p=0.00011
Gene significance for Pronephros
Module Membership in grey module
B
Module membership vs. gene significance
cor=0.41, p=4.6e−13
Gene significance for Brain
Module Membership in turquoise module
C
Module membership vs. gene significance
cor=0.71, p=3.1e−44
Gene significance for Muscle
Module Membership in blue module
D
Module membership vs. gene significance
cor=0.78, p=4.8e−09
Gene significance for Muscle
Module Membership in yellow module
