# Supplementary material for: Expression Profiles of Housekeeping Genes and Tissue-Specific Genes in Different Tissues of Chinese Sturgeon (Acipenser sinensis)
Source: Animals (Basel). 2024 Nov 21;14(23):3357. doi: 10.3390/ani14233357 (PMC11639794; doi:10.3390/ani14233357)
Supplement: Supplementary file 1 [file animals-14-03357-s001.zip › Figure S7.pdf]

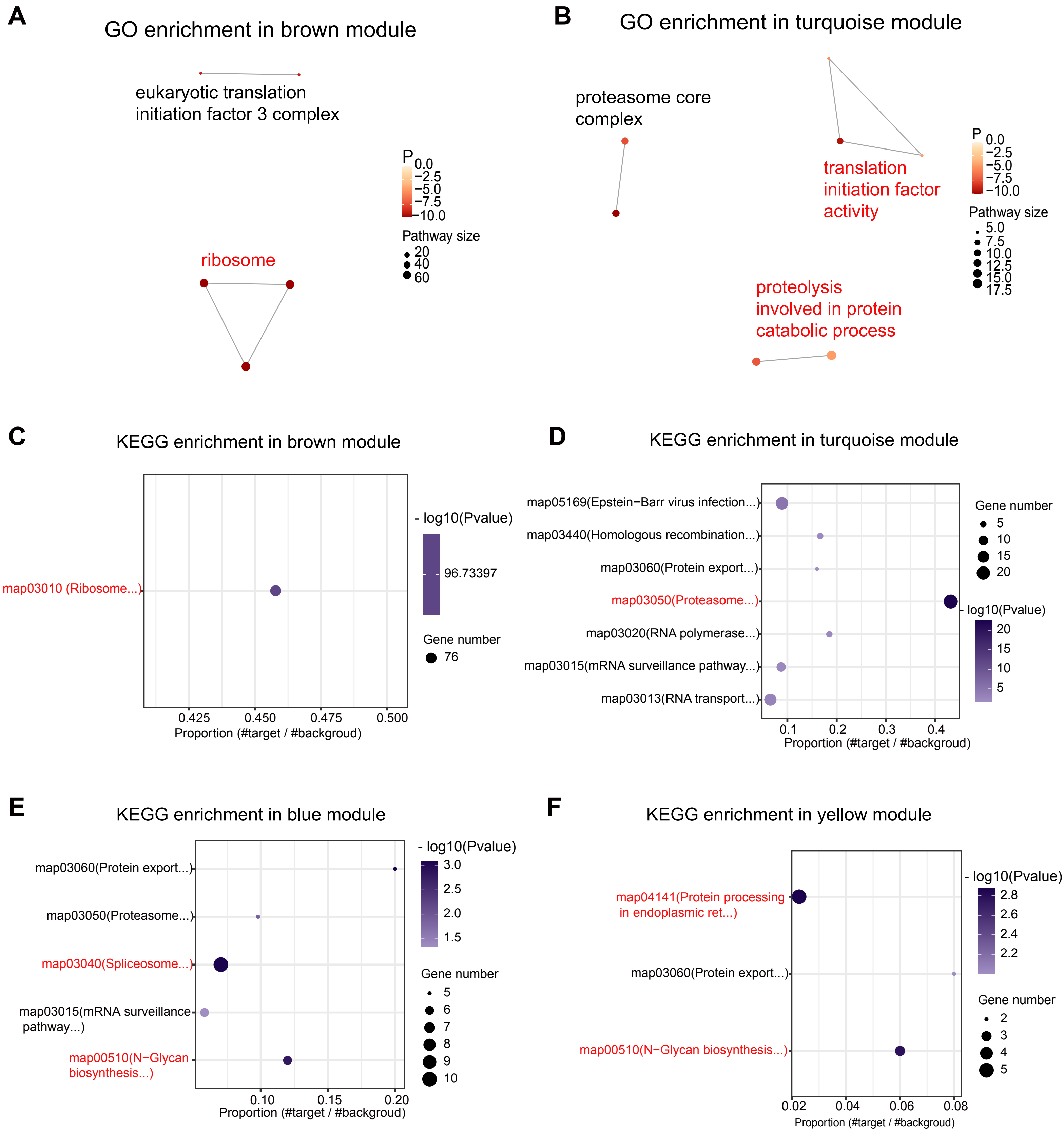

A
GO enrichment in brown module
eukaryotic translation initiation factor 3 complex
ribosome
P
0.0
−2.5
−5.0
−7.5
−10.0
Pathway size
20
40
60
B
GO enrichment in turquoise module
proteasome core complex
translation initiation factor activity
proteolysis involved in protein catabolic process
P
0.0
−2.5
−5.0
−7.5
−10.0
Pathway size
5.0
7.5
10.0
12.5
15.0
17.5
C
KEGG enrichment in brown module
map03010 (Ribosome...)
0.425
0.450
0.475
0.500
Proportion (#target / #backgroud)
- log10(Pvalue)
96.73397
Gene number
76
D
KEGG enrichment in turquoise module
map05169(Epstein−Barr virus infection...)
map03440(Homologous recombination...)
map03060(Protein export...)
map03050(Proteasome...)
map03020(RNA polymerase...)
map03015(mRNA surveillance pathway...)
map03013(RNA transport...)
0.1
0.2
0.3
0.4
Proportion (#target / #backgroud)
Gene number
5
10
15
20
- log10(Pvalue)
20
15
10
5
E
KEGG enrichment in blue module
map03060(Protein export...)
map03050(Proteasome...)
map03040(Spliceosome...)
map03015(mRNA surveillance pathway...)
map00510(N−Glycan biosynthesis...)
0.10
0.15
0.20
Proportion (#target / #backgroud)
- log10(Pvalue)
3.0
2.5
2.0
1.5
Gene number
5
6
7
8
9
10
F
KEGG enrichment in yellow module
map04141(Protein processing in endoplasmic ret...)
map03060(Protein export...)
map00510(N−Glycan biosynthesis...)
0.02
0.04
0.06
0.08
Proportion (#target / #backgroud)
- log10(Pvalue)
2.8
2.6
2.4
2.2
Gene number
2
3
4
5
