# Supplementary material for: Expression Profiles of Housekeeping Genes and Tissue-Specific Genes in Different Tissues of Chinese Sturgeon (Acipenser sinensis)
Source: Animals (Basel). 2024 Nov 21;14(23):3357. doi: 10.3390/ani14233357 (PMC11639794; doi:10.3390/ani14233357)
Supplement: Supplementary file 1 [file animals-14-03357-s001.zip › Supplementary Materials.pdf]

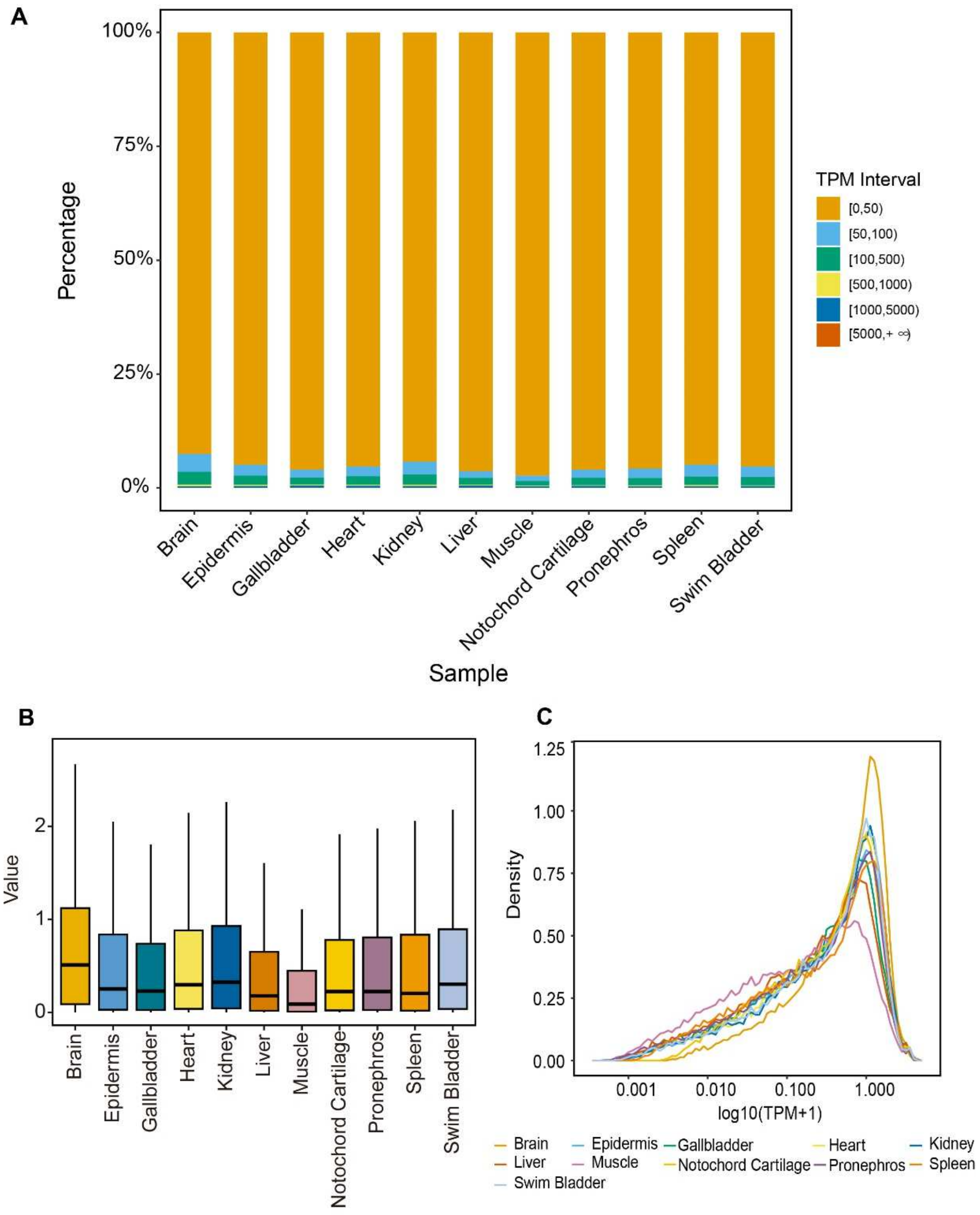

Figure S1. Gene expression level analysis. **(A)** TPM interval distribution in different tissues. **(B)** TPM box plot. **(C)** TPM density distribution.

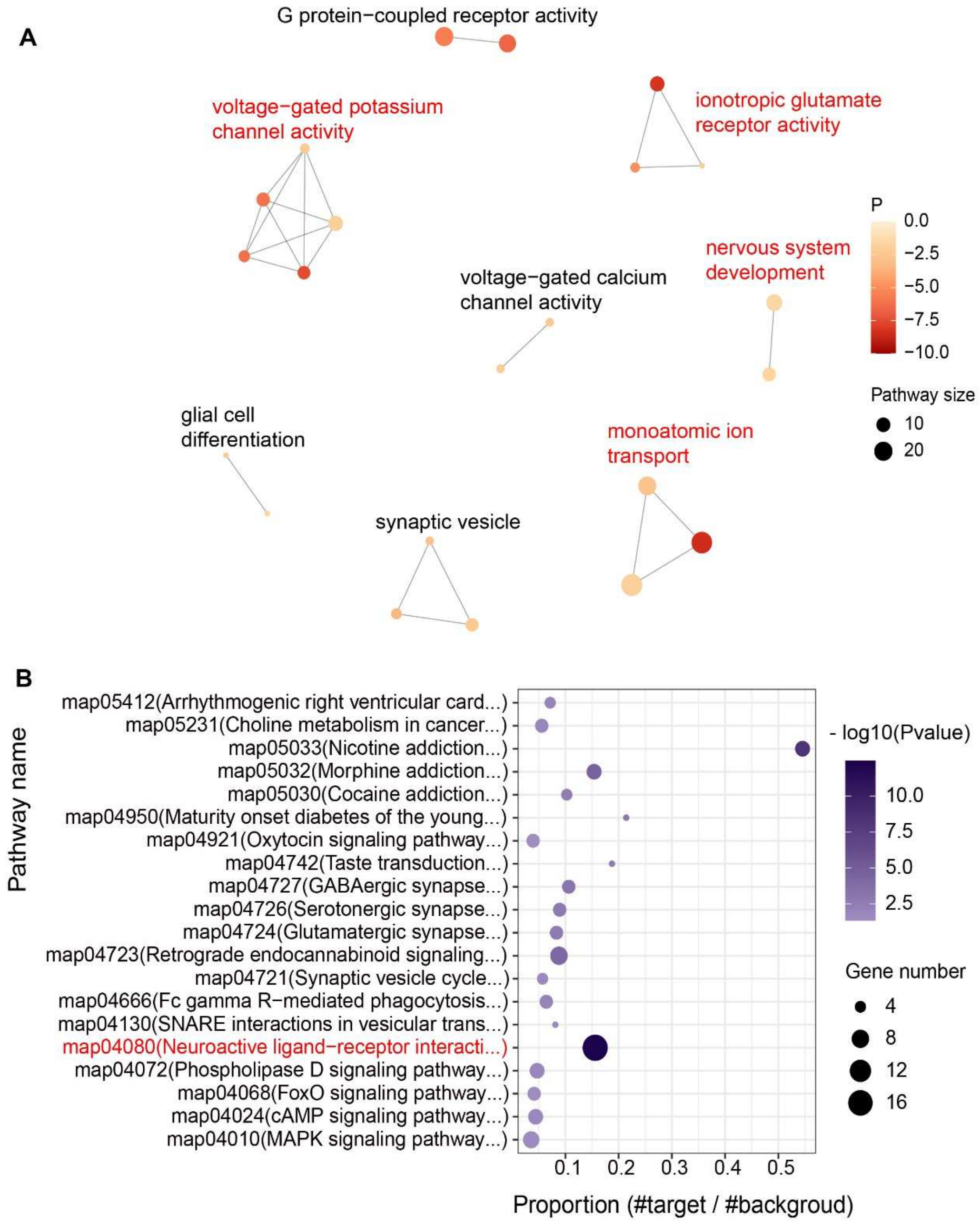

Figure S2. Specific gene enrichment in brain. **(A)** Network diagram illustrating the significantly enriched GO terms in brain. **(B)** Bubble chart of significantly enriched KEGG pathways in brain.

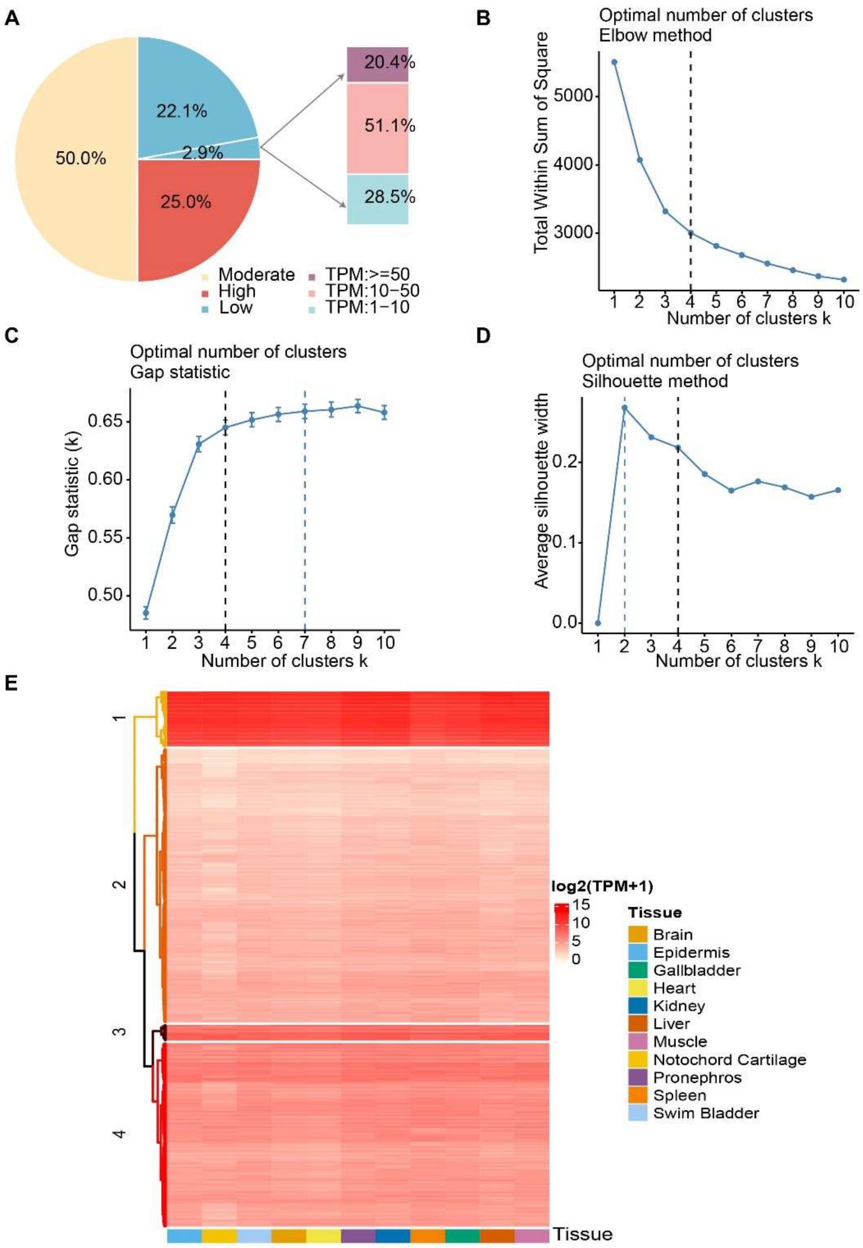

Figure S3. Coefficient of variation, optimal number of K-means clusters, and heat map of gene expression for housekeeping genes. **(A)** Pie chart of coefficient of variation and housekeeping genes. The figure illustrated distinct levels of variation within different groups, with red, yellow, and blue denoting high, moderate, and low variation groups, respectively. The blue segment specifically indicated the percentage of housekeeping genes within the dataset. By categorizing housekeeping genes based on their TPM values, the expression levels were further segmented into purple, pink, and blue categories, representing genes with TPM values exceeding 50, those falling between 10 and 50, and those ranging from 1 to 10, respectively. **(B-D)** The optimal number of K-means clusters for housekeeping genes obtained based on the Elbow method, Silhouette method, and Gap statistic, respectively. **(E)** Heat map of gene expression in housekeeping genes.

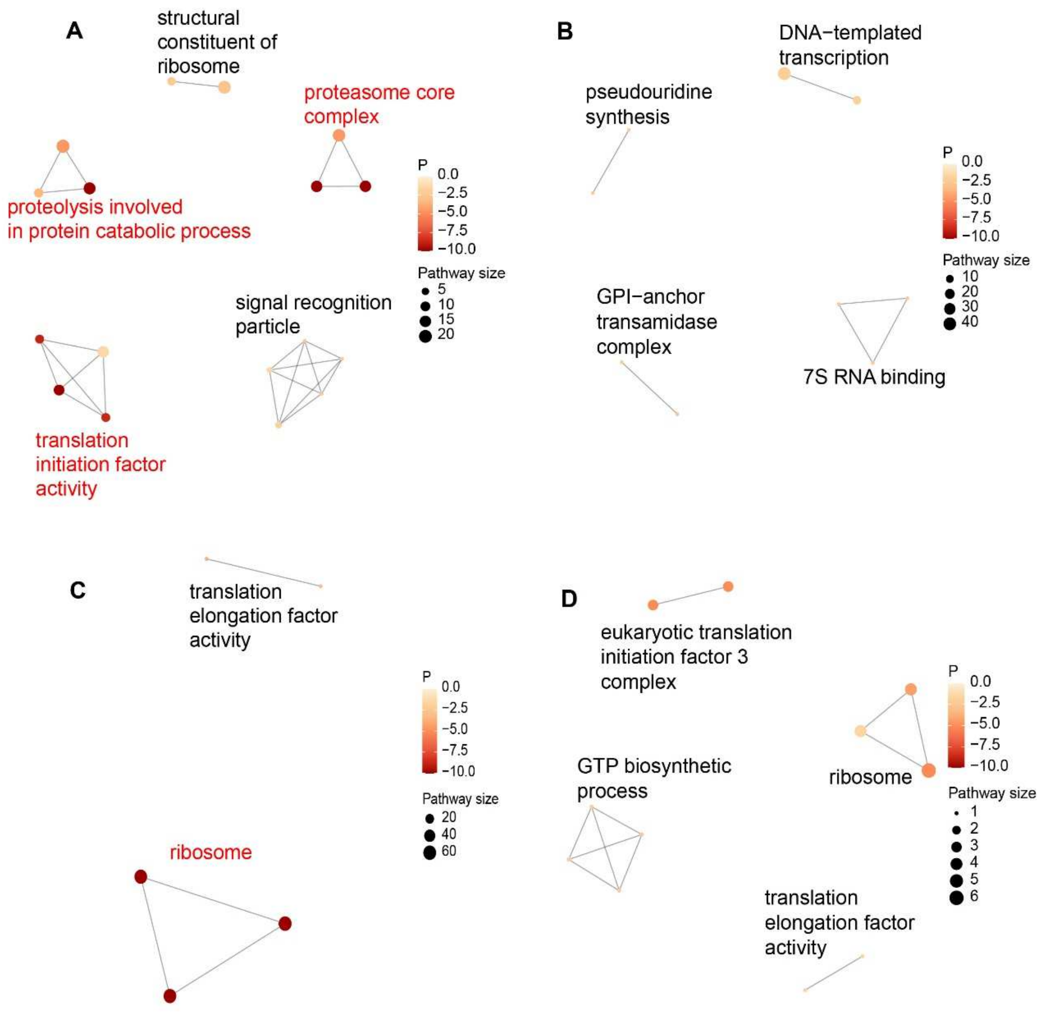

Figure S4. Network diagram of significantly enriched GO terms in various clusters of housekeeping genes. **(A-D)** Network diagram of significantly enriched GO terms from clusters 1 to cluster 4 of housekeeping genes.

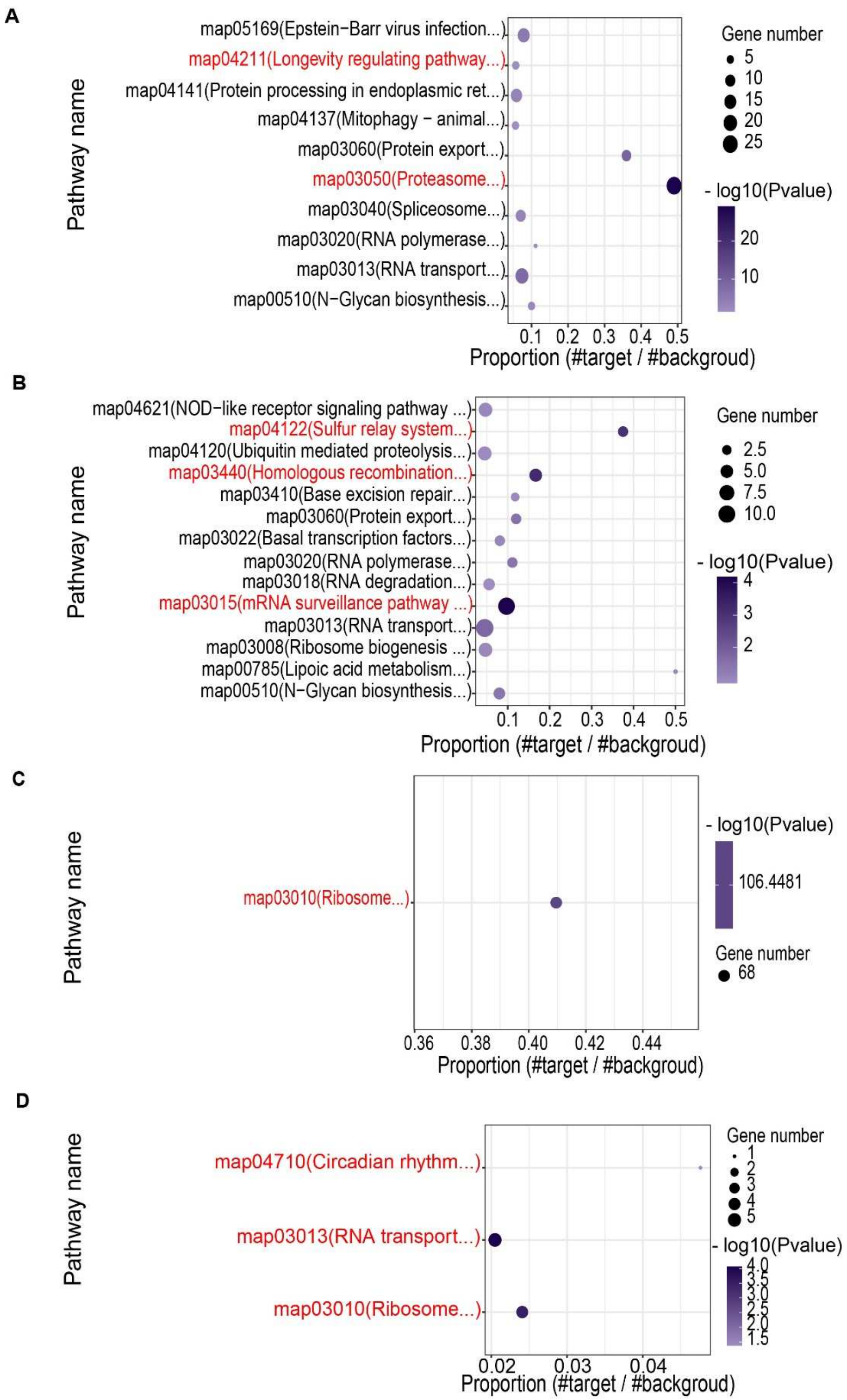

Figure S5. Bubble chart of significantly enriched KEGG pathways in various clusters of housekeeping genes. **(A-D)** Bubble chart of significantly enriched KEGG pathways from clusters 1 to cluster 4 of housekeeping genes.

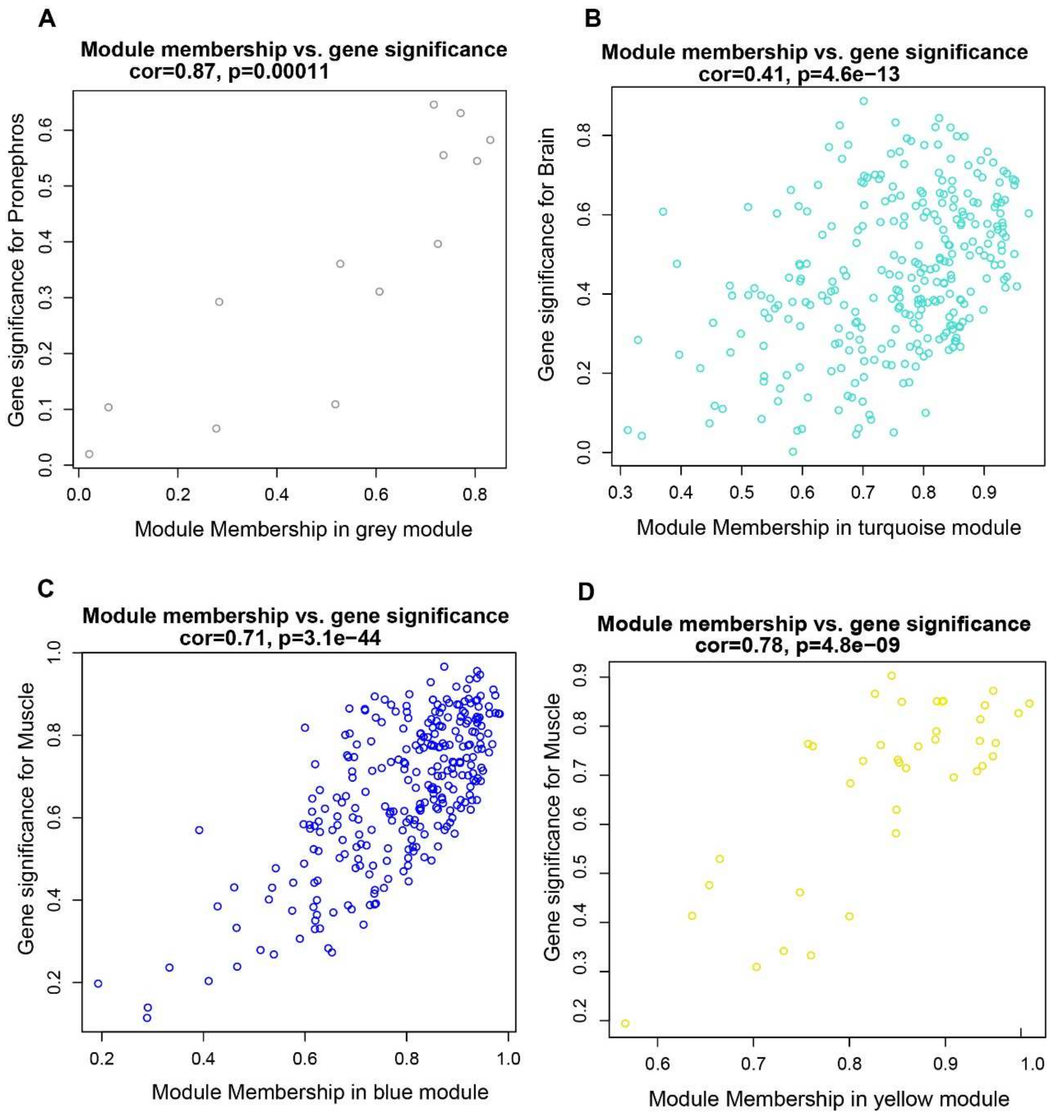

Figure S6. Scatterplot of gene significance for tissues versus module membership in the partial selected module. **(A)** a scatterplot of gene significance for brain versus module membership in the turquoise module. **(B)** a scatterplot of gene significance for pronephros versus module membership in the grey module. **(C)** a scatterplot of gene significance for muscle versus module membership in the blue module. **(D)** a scatterplot of gene significance for muscle versus module membership in the yellow module.

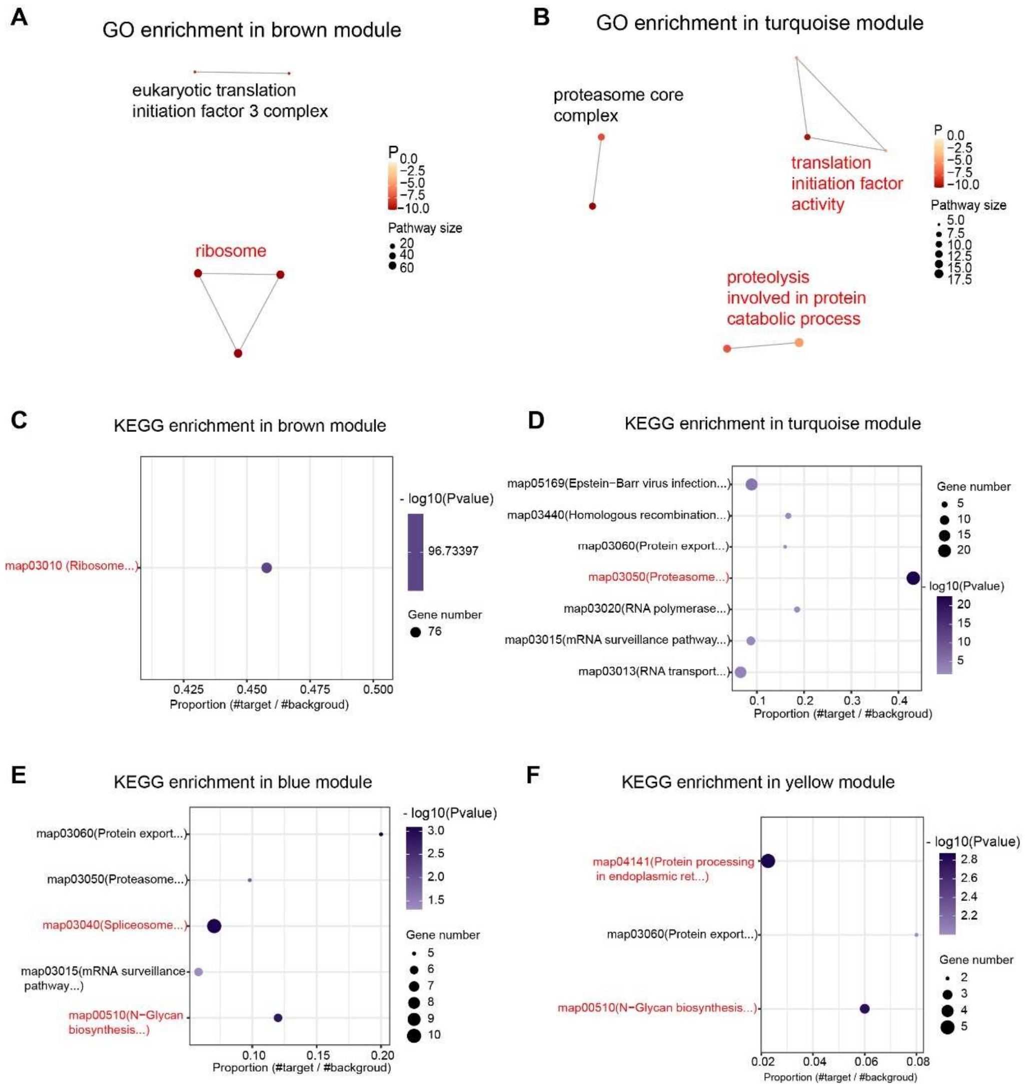

Figure S7. Results of significant enrichment based on the GO and KEGG databases in each module. **(A-B)** GO enrichment results for brown and turquoise modules, respectively. **(C-F)** Significantly enriched pathways in the KEGG analysis of brown, turquoise, blue, and yellow modules, respectively.
