# Supplementary figures and images for: Expression Profiles of Housekeeping Genes and Tissue-Specific Genes in Different Tissues of Chinese Sturgeon (Acipenser sinensis)
Source: Animals (Basel). 2024 Nov 21;14(23):3357. doi: 10.3390/ani14233357 (PMC11639794; doi:10.3390/ani14233357)

**A**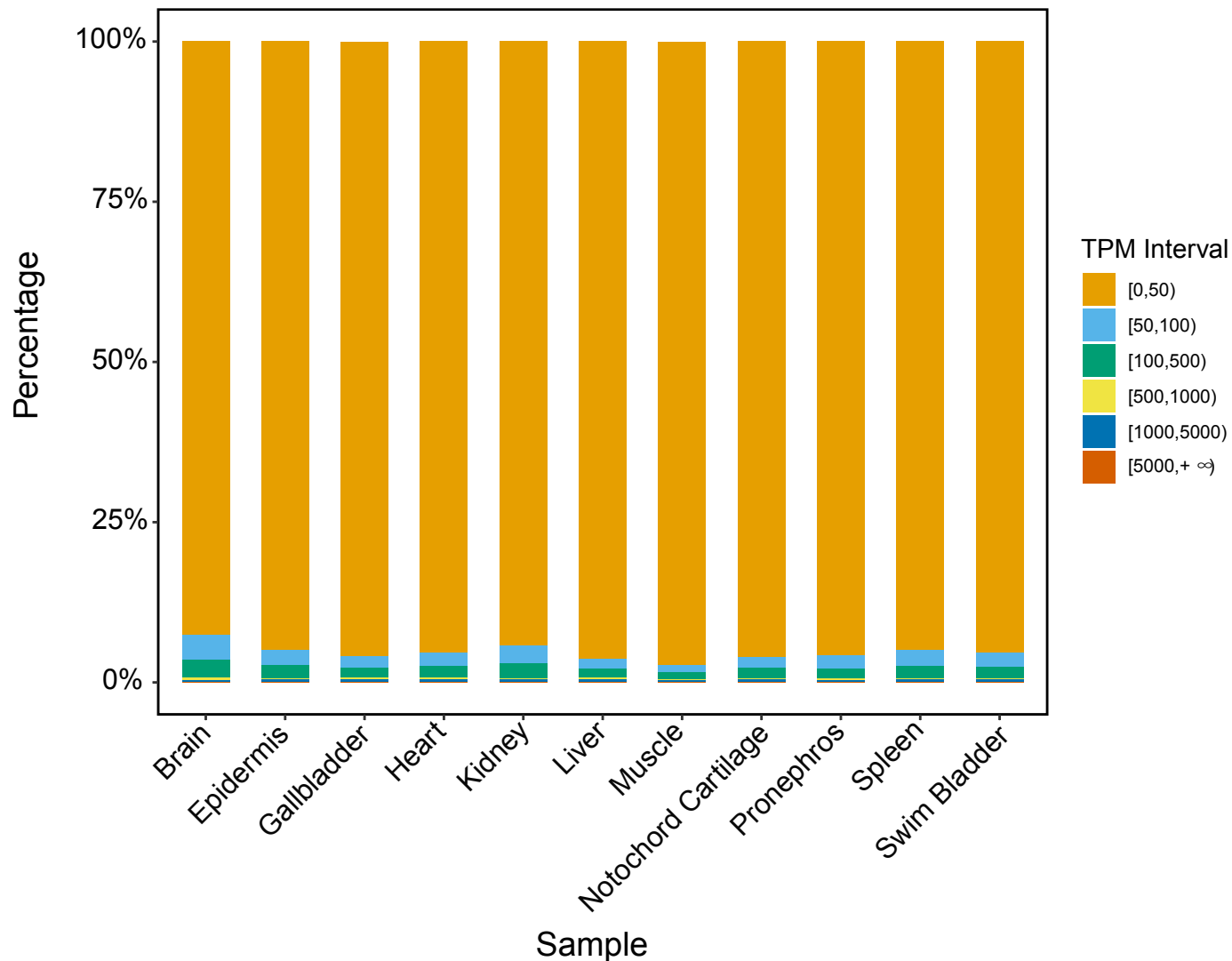**B**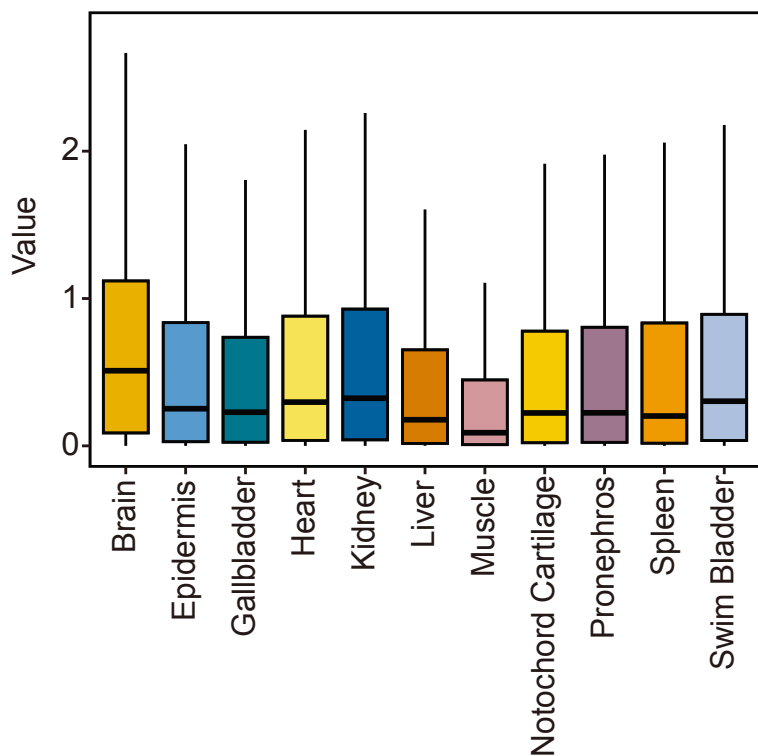**C**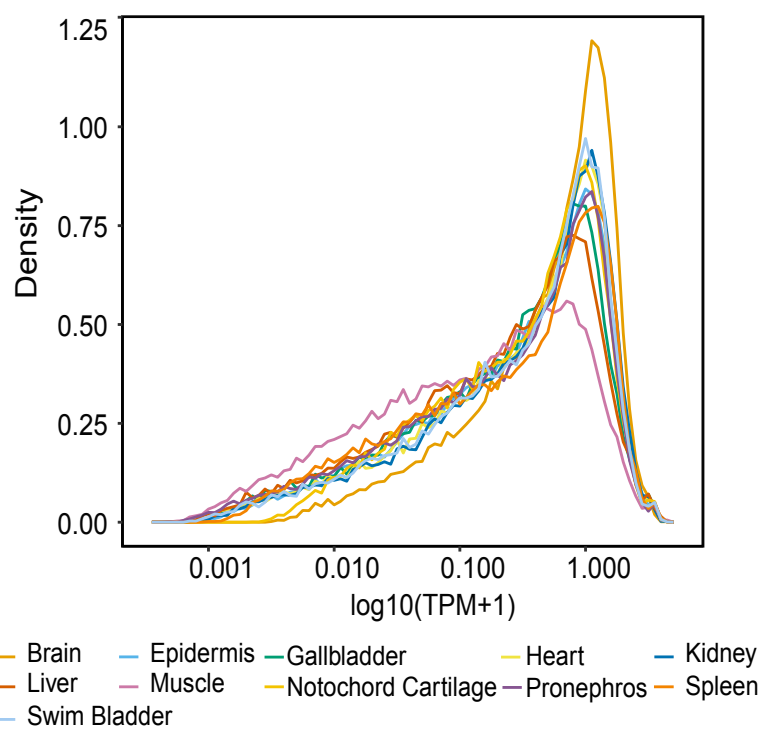

Supplement: Supplementary file 1 [file animals-14-03357-s001.zip › Figure S1.pdf]

**A**

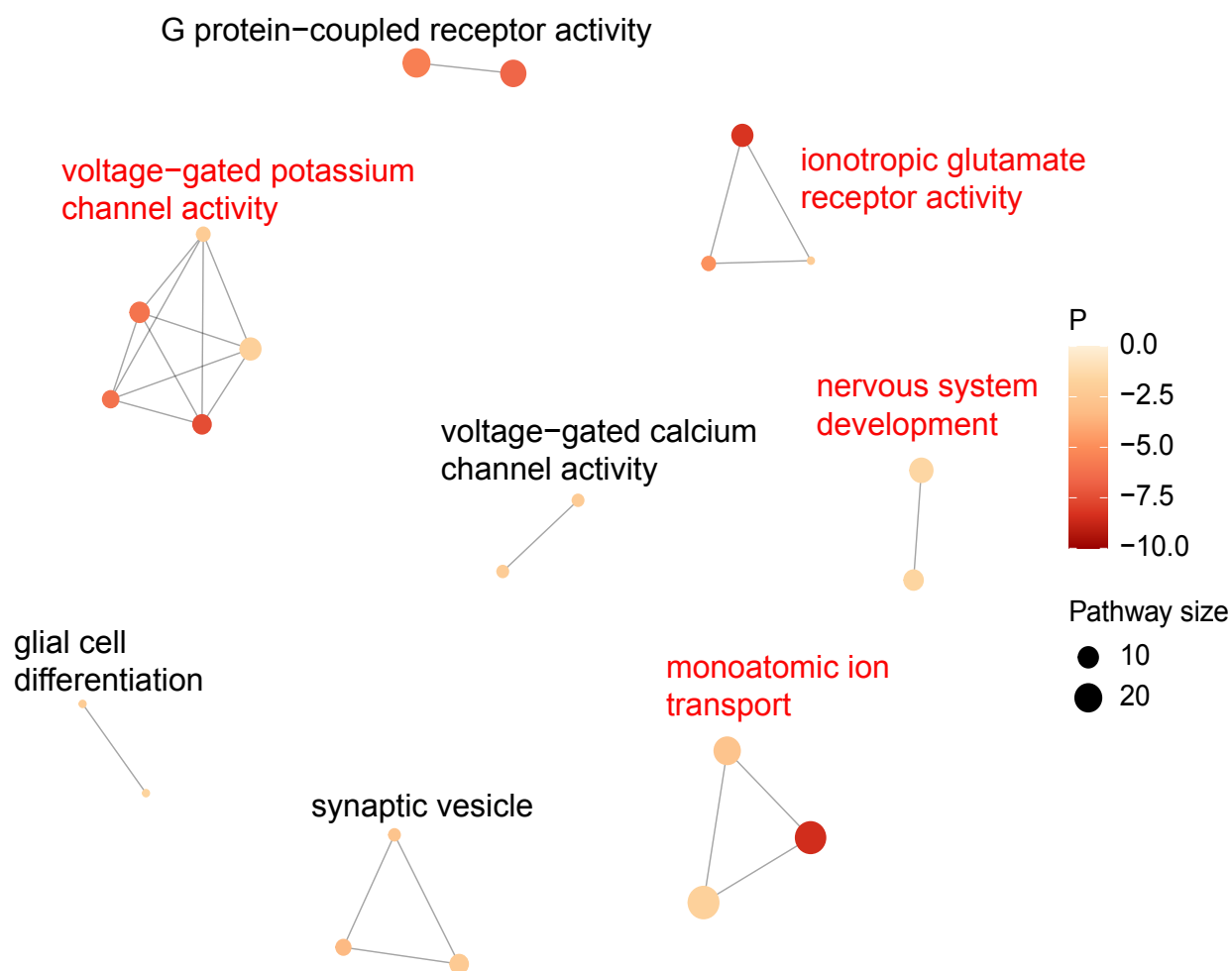

**B**

Pathway name

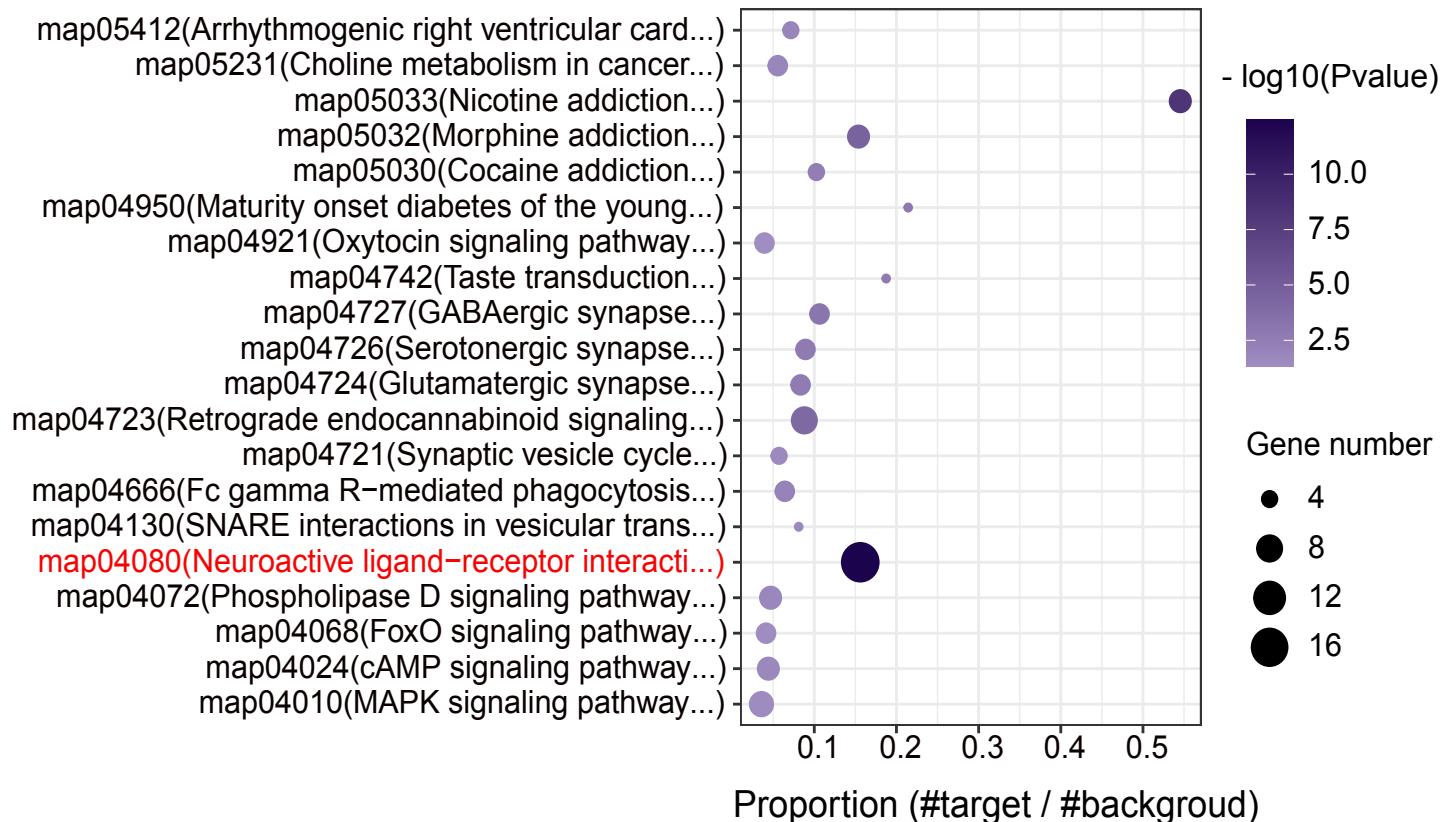

Supplement: Supplementary file 1 [file animals-14-03357-s001.zip › Figure S2.pdf]

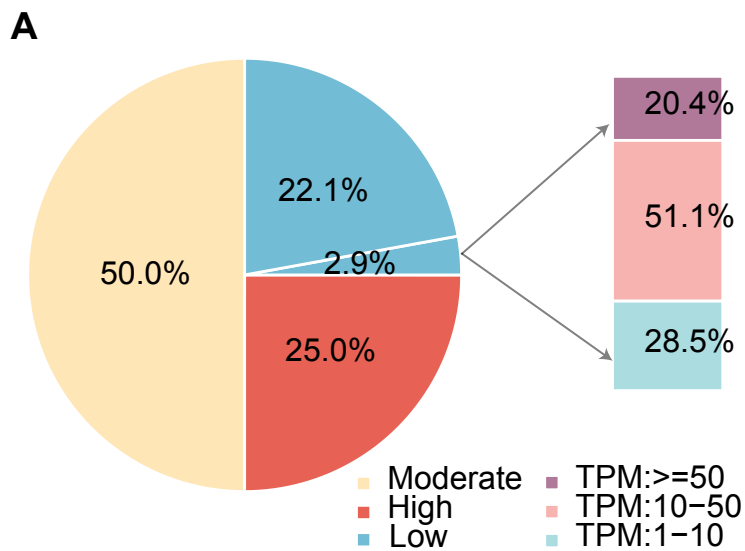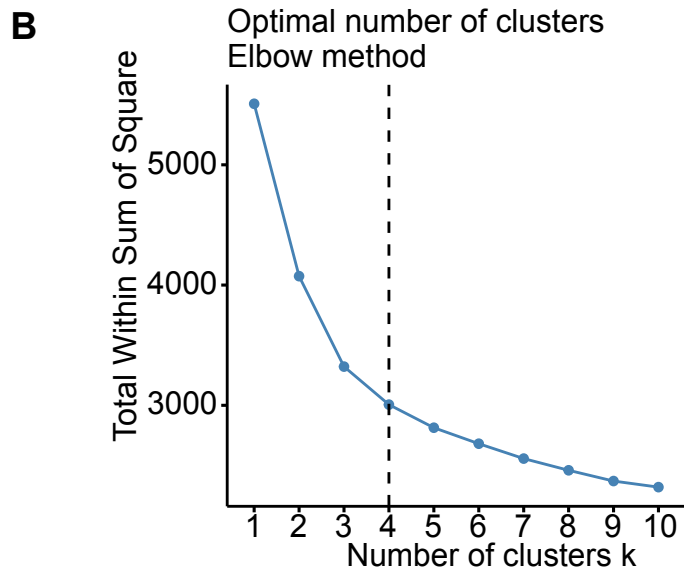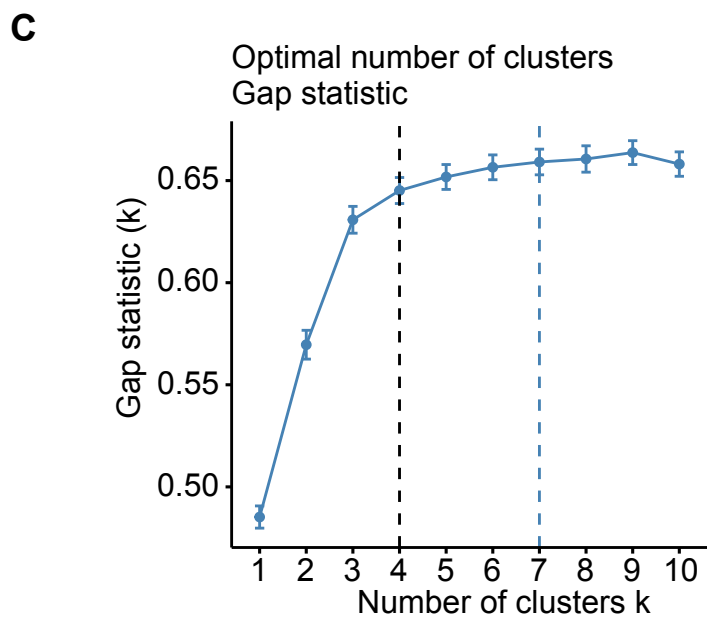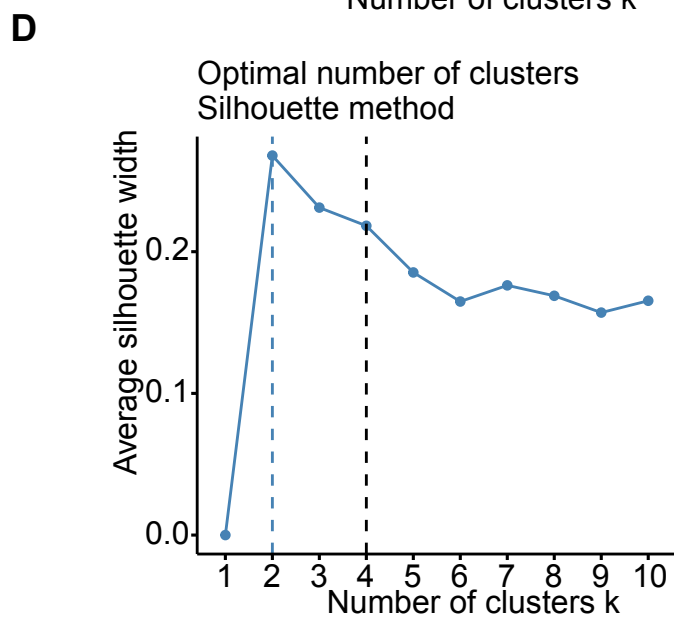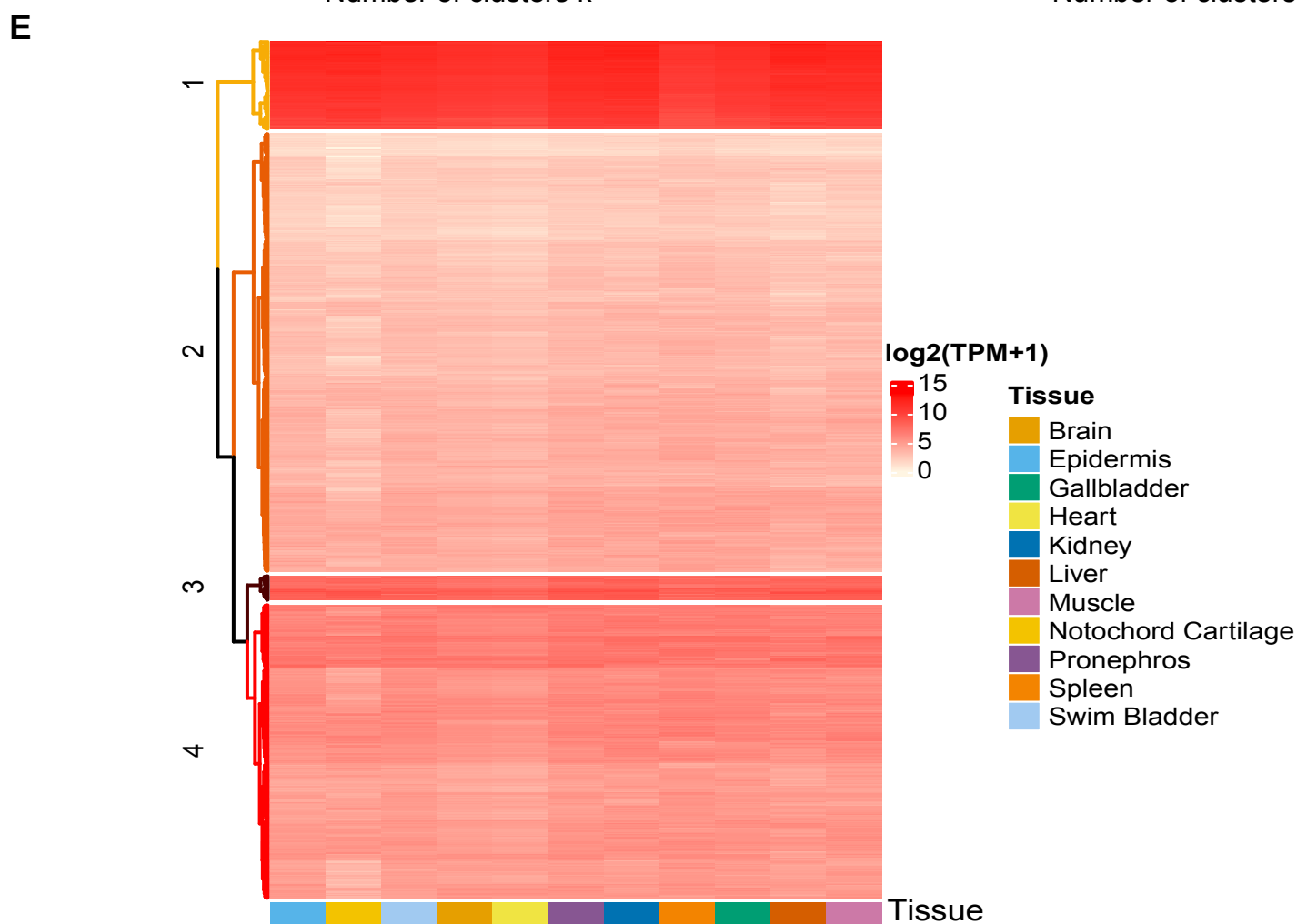

Supplement: Supplementary file 1 [file animals-14-03357-s001.zip › Figure S3.pdf]

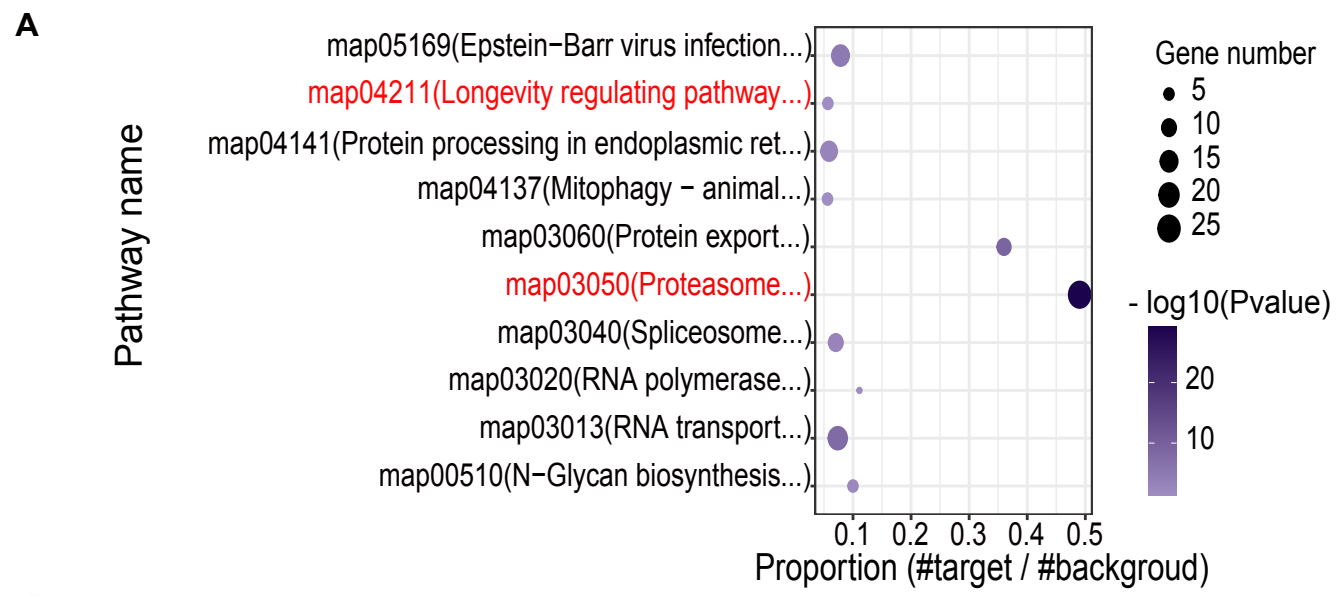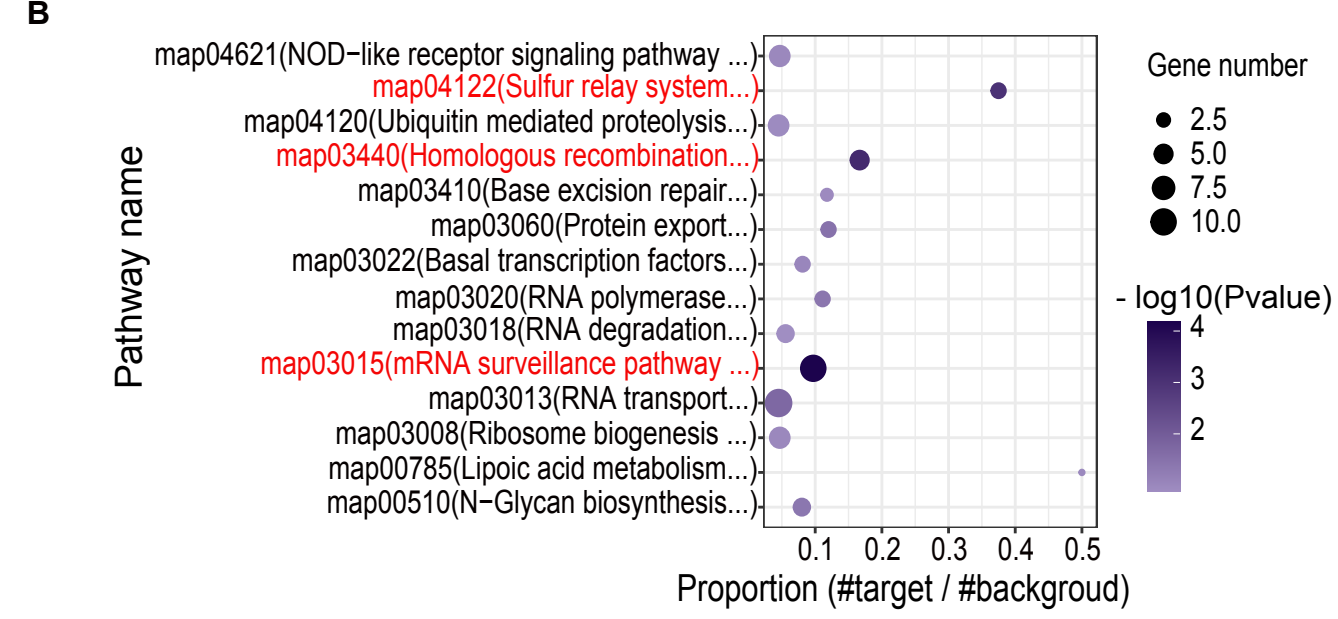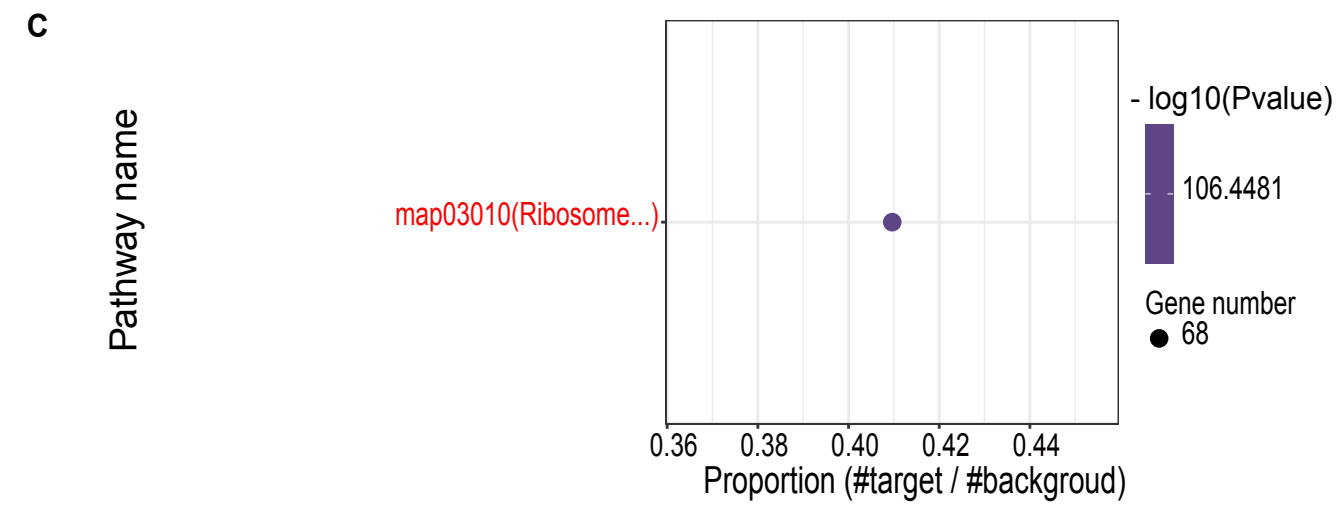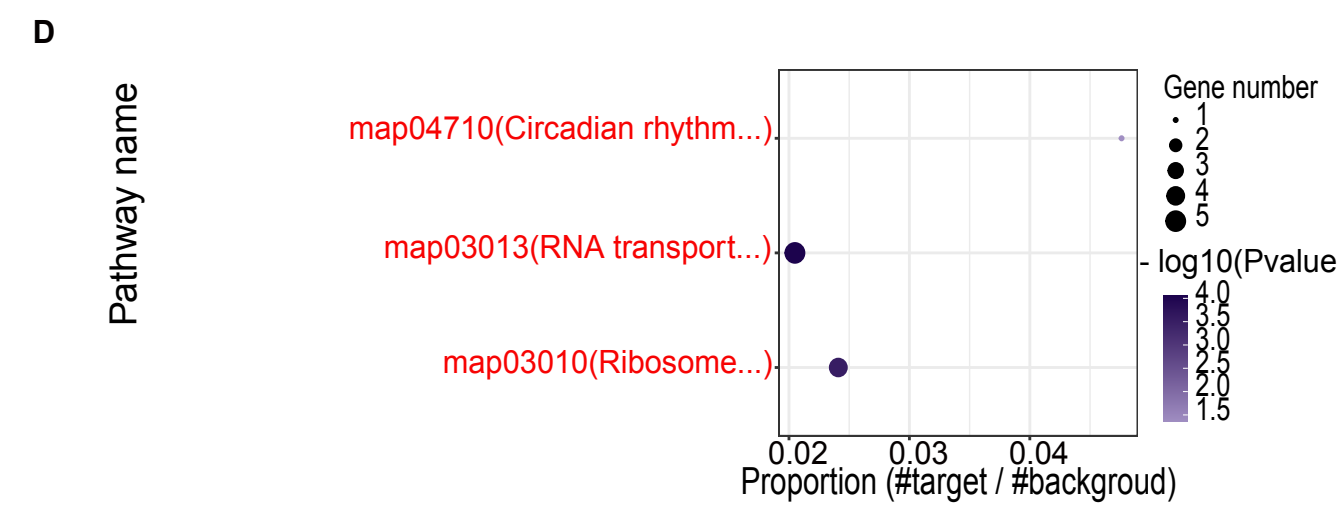

Supplement: Supplementary file 1 [file animals-14-03357-s001.zip › Figure S5.pdf]
